# Delayed-Onset Fibrotic Capsular Bag Distension Syndrome

**DOI:** 10.7759/cureus.104065

**Published:** 2026-02-22

**Authors:** Tarannum Mansoori, Mantravadi L Karthika

**Affiliations:** 1 Ophthalmology, Prime Retina Eye Care Center, Hyderabad, IND; 2 Ophthalmology, Anand Eye Institute, Hyderabad, IND

**Keywords:** capsular bag distension, capsular bag distension syndrome, induced myopia, intraocular lens, iol (intraocular lens)

## Abstract

Capsular bag distension syndrome (CBDS) is an uncommon complication of cataract surgery following in-the-bag posterior chamber intraocular lens (PCIOL) implantation. It is characterized by the accumulation of turbid fluid between the IOL optic and the posterior capsule, frequently resulting in reduced visual acuity and a myopic refractive shift. The late-onset fibrotic variant is particularly rare and may manifest several years after otherwise uneventful surgery.

A 52-year-old man presented with progressive visual blurring 10 years after uncomplicated phacoemulsification with in-the-bag PCIOL implantation. Slit-lamp biomicroscopy demonstrated turbid fluid trapped between the IOL optic and the posterior capsule. Anterior segment optical coherence tomography confirmed the diagnosis of CBDS. Neodymium-doped yttrium aluminium garnet (Nd:YAG) posterior capsulotomy resulted in immediate evacuation of the accumulated fluid; however, a persistent retrolenticular space and residual myopic shift were observed.

In conclusion, very-late-onset fibrotic CBDS should be considered in patients presenting with unexplained refractive changes or retro-IOL fluid accumulation years after cataract surgery. This case underscores an unusually delayed presentation occurring a decade postoperatively. The persistent myopic shift suggests incomplete refractive reversibility, which is typically expected following Nd:YAG laser capsulotomy. This sustained refractive alteration is most plausibly attributable to the continued presence of a retrolenticular space, secondary to retained temporal cortical material, which maintained capsular bag patency despite successful drainage of the turbid fluid.

## Introduction

Capsular bag distension syndrome (CBDS) [1], also referred to as capsular block syndrome, capsular bag hyperdistension, or capsulorhexis block syndrome, is an uncommon complication of cataract surgery [2] following in-the-bag posterior chamber intraocular lens (PCIOL) implantation. It is characterized by the accumulation of clear or turbid, milky fluid within the capsular bag, specifically between the IOL optic and the posterior capsule. This fluid collection results in anterior axial displacement of the IOL, typically manifesting as a myopic refractive shift and decreased visual acuity after cataract extraction [3].

The incidence of CBDS is relatively low, with reported rates ranging from approximately 0.3% to 1.6% [4], mostly reflecting early post-operative cases, among patients undergoing phacoemulsification with PCIOL implantation following cataract surgery. Clinical presentation is variable, with early-onset cases occurring within weeks to months postoperatively [5,6], and delayed manifestations reported several years after the initial procedure [7,8].

We report a case of late-onset CBDS presenting 10 years after an uneventful cataract surgery, in which both the myopic shift and the posterior capsular space persisted despite successful neodymium-doped yttrium aluminium garnet (Nd:YAG) capsulotomy and evacuation of the entrapped fluid.

## Case presentation

**Figure 1:**
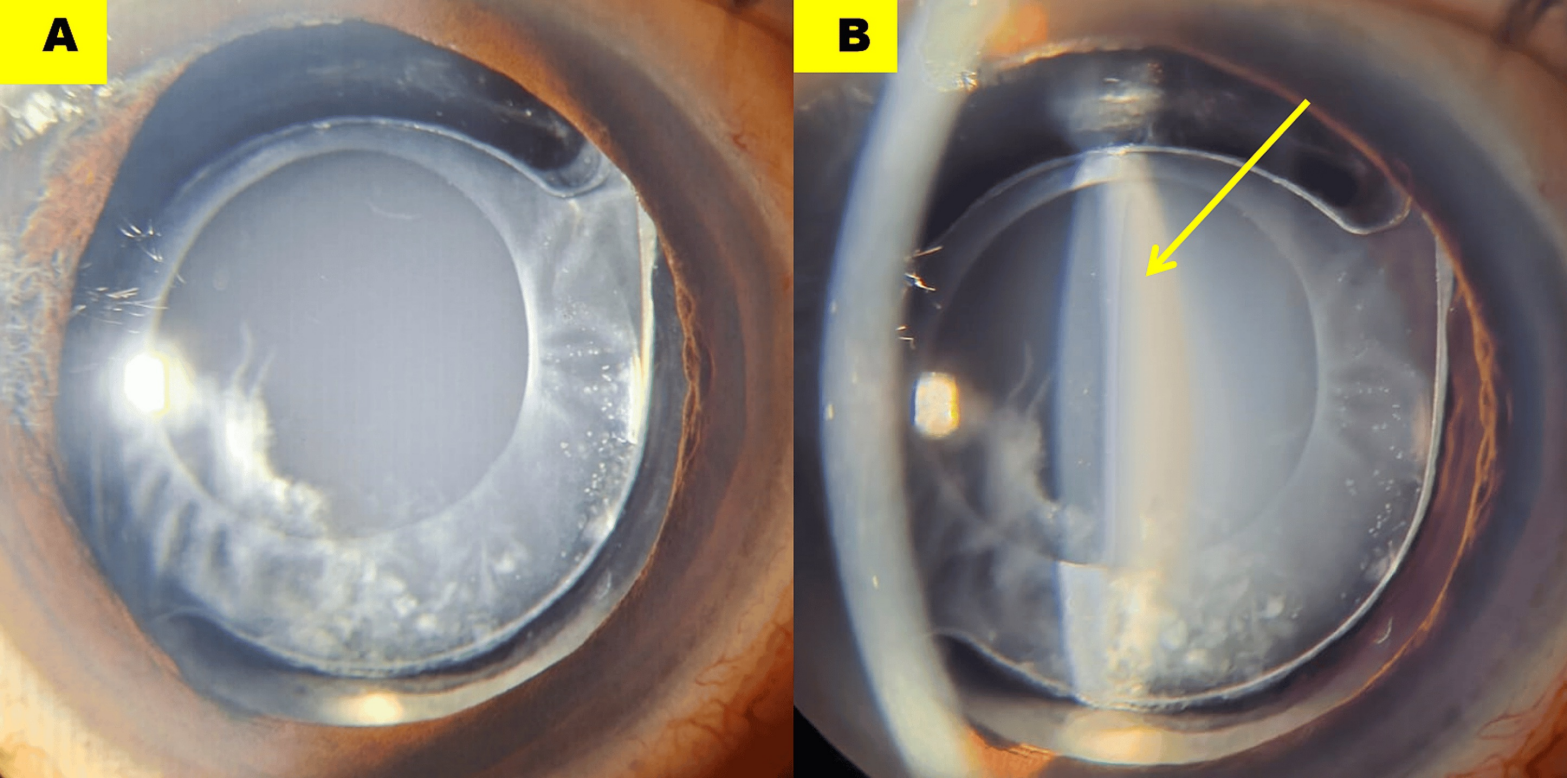
Slit-lamp photographs of diffuse (A) and slit (B) view demonstrating a pocket of turbid fluid (B, yellow arrow) sequestered between the posterior capsule and the intraocular lens optic

A 52-year-old man presented with blurred vision in the right eye, 10 years after an uneventful phacoemulsification with in-the-bag PCIOL implantation. His best-corrected visual acuity was 20/20 p with -2.00 diopters (D) spherical correction, and the intraocular pressure was 14 mm Hg. Slit-lamp examination revealed a pocket of turbid, smoky fluid entrapped between the posterior capsule and the PCIOL (Figures 1A, 1B; yellow arrow).

**Figure 2:**
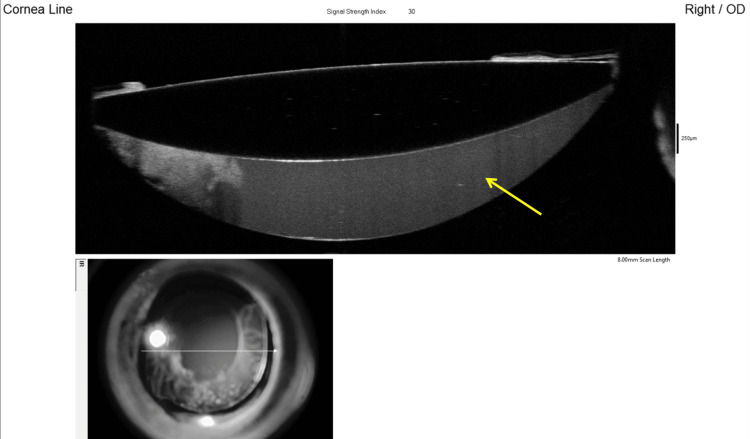
Anterior segment optical coherence tomography shows a well-defined hyperreflective compartment (yellow arrow) located posterior to the intraocular lens

Anterior segment optical coherence tomography (AS-OCT) demonstrated a well-defined hyperreflective space, delineating a separation between the IOL optic and the posterior capsule (Figure 2; yellow arrow).

**Figure 3:**
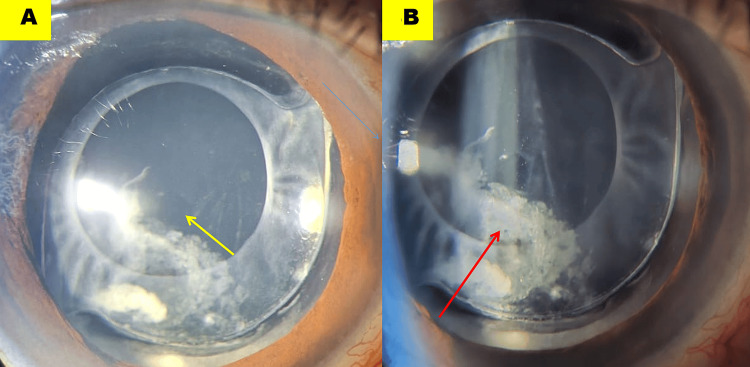
Neodymium-doped yttrium aluminium garnet posterior capsulotomy (A, yellow arrow) facilitated release of the entrapped fluid. A residual retro-intraocular lens space persisted due to retained cortical material, most prominent temporally (B, red arrow).

A diagnosis of CBDS was made, and using a neodymium-doped yttrium aluminium garnet (Nd:YAG) laser, central posterior capsulotomy was performed. The CBDS resolved immediately following the release of the turbid fluid through the capsulotomy (Figure 3A; yellow arrow).

**Figure 4:**
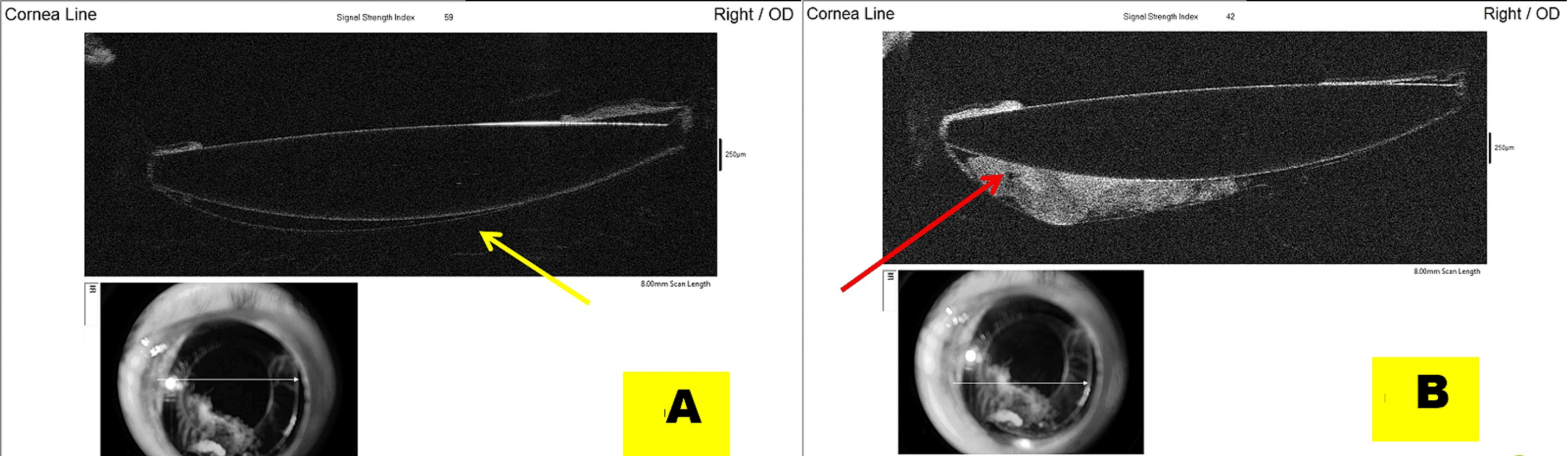
Anterior segment optical coherence tomography shows that the residual retro-intraocular lens space decreased in size but remains evident (A, yellow arrow), accounting for the persistent myopic shift despite decompression. Retained cortical material, most prominent temporally, contributes to maintenance of this space (B, red arrow).

The myopic shift also remained due to the persistent retro-IOL space (Figures 4A, 4B), as confirmed on AS-OCT.

Postoperatively, the patient was treated with 1% prednisolone acetate eye drops administered every two hours for one week. He showed both clinical and symptomatic improvement. One week later, the best-corrected visual acuity was 20/20 with a -2.00 D spherical correction.

## Discussion

The first documented case of CBDS was reported in 1990 [9], and the term “capsular block syndrome” was subsequently introduced in 1993 [2].

The precise mechanism underlying this phenomenon remains incompletely understood, and several hypotheses have been proposed. One prevailing theory suggests that capsular distention results from the influx of aqueous humor into the capsular bag driven by an osmotic gradient across the capsule. This gradient may be established by retained viscoelastic material within the bag, which facilitates the intraoperative accumulation and entrapment of fluid [10].

The late or fibrotic variant [3] is attributed to proliferation or metaplastic transformation of residual lens epithelial cells, or to retained cortical material, which can create a sealed compartment that resists simple decompression.

In late fibrotic CBDS, adhesion of the anterior capsulotomy rim to the IOL optic may impede the normal egress of metabolic fluid. This fibrotic phase develops in the late postoperative period and is driven by residual lens epithelial cells that undergo metaplasia and proliferate, producing collagen and extracellular matrix [8] that accumulate within the capsular bag. Over time, the trapped fluid may become turbid or increasingly proteinaceous, while mild anterior displacement of the IOL contributes to an associated myopic shift.

In the present case, the markedly delayed onset, occurring a decade after cataract surgery, together with the persistence of a myopic shift despite laser decompression, strongly supports a fibrotic subtype. This case is noteworthy because, although the patient’s blurred vision resolved, the retrolenticular space persisted, reflecting the underlying fibrotic component of retained cortical material. This suggests that residual cortical material maintained a partially sealed compartment even after Nd:YAG capsulotomy successfully drained the milky fluid.

Recurrent or refractory cases have been documented, occasionally necessitating surgical intervention, such as capsular bag lavage or aspiration of the retained material [7], when Nd:YAG laser capsulotomy alone proves insufficient.

The most effective preventive strategy for CBDS is the meticulous intraoperative removal of ophthalmic viscoelastic devices. This entails comprehensive aspiration of all residual viscoelastic material from the capsular bag, combined with thorough cortical cleanup and careful anterior and posterior capsular polishing. This minimizes retained substances within the capsular bag and thereby significantly reduces the risk of postoperative capsular distension.

Close monitoring is essential during the early postoperative period for any atypical inflammatory responses. Although rare, cases of Propionibacterium acnes-associated endophthalmitis have been reported following Nd:YAG capsulotomy. This complication is thought to result from the release of a sequestered, low-virulence organism into the vitreous during the procedure [11].

## Conclusions

Very-late-onset fibrotic CBDS represents a distinctive postoperative entity in which long asymptomatic intervals may mask a chronically evolving, compartmentalized capsular process. Clinicians should maintain a high index of suspicion for this diagnosis when encountering unexplained refractive shifts or retro-IOL fluid collections, even many years after surgery. Persistent myopic shift after Nd:YAG capsulotomy should prompt consideration of an underlying fibrotic process, rather than being attributed solely to incomplete decompression.
